# Electrophysiological Investigation of Different Methods of Anesthesia in Lobster and Crayfish

**DOI:** 10.1371/journal.pone.0162894

**Published:** 2016-09-19

**Authors:** Torsten Fregin, Ulf Bickmeyer

**Affiliations:** Alfred Wegener Institut – Helmholtz Zentrum für Polar- und Meeresforschung, Am Handelshafen 12, 27570, Bremerhaven, Germany; Universitat Wursburg, GERMANY

## Abstract

**Objectives:**

In search for methods of anesthesia of crustaceans, an implanted electrode into lobster and crayfish CNS enabled us to monitor signal propagation in the nerve system of animals undergoing different protocols.

**Results:**

Cooling (tap water 0°C, sea water -1,8°C) and anesthesia with MgCl_2_ (10%) were both discarded as anesthetic procedures because responses to external stimuli were still detectable under treatment. Contrarily, bubbling the aquarium water with CO_2_ can be considered a “partially successful” anesthesia, because signal propagation is inhibited but before that the animals show discomfort. The procedure of “electro-stunning” induces epileptic-form seizures in the crustacean CNS (lobster, crayfish), which overlay but do not mitigate the response to external stimuli. After several minutes the activity declines before the nervous system starts to recover. A feasible way to sacrifice lobsters is to slowly raise the water temperature (1°C min^-1^), as all electrical activities in the CNS cease at temperatures above ~30°C, whereas below this temperature the animals do not show signs of stress or escape behavior (e.g. tail flips) in the warming water.

**Conclusion:**

CO_2_ is efficient to anaesthetize lobster and crayfish but due to low pH in water is stressful to the animals previous to anesthesia. Electrical stunning induces epileptiform seizures but paralyses the animals and leads to a reversible decline of nerve system activity after seizure. Electric stunning or slowly warming just before preparation may meet ethical expectations regarding anaesthesia and to sacrifice crustaceans.

## Introduction

Besides for human consumption [1], (higher) crustaceans are used for scientific experiments (e.g. [2]). More than fifty years ago, Baker (1955) and Gunter (1960) proposed methods for “ethic” or “painless” killing of crabs and crayfish [3,4], which initiated a debate in the scientific community [5–8]. Some of the proposed methods for stunning, anaesthesia and killing were recently tested in crabs [9], but did not include direct measurements in the central nervous system. Crustaceans are well-established models for neurobiological research [10,11]. The large decapod crustaceans (lobsters and shrimp) are particularly well studied with respect to sensory and neuronal circuits (e.g. [12]) and hormonal (e.g. [13]) or neuro-modulatory aspects (e.g.[14,15]). Studies reporting experimental research often provide succinct statements concerning preparative methods such as “animals were anesthetized on ice for 30 to 45 min” or “the nervous system was dissected out”, without indicating whether a state of anaesthesia had indeed been reached.

Recently it has been shown, that the crustacean nervous system is capable of preserving nerve cell communication and rhythmicity of pattern generators stably even at extremely low temperatures [16–18]. Therefore, it is well possible that cooling only reduces the basal metabolic rate (and turns muscles “stiff”), while the animals are still able to compute sensory information. This prompted us to question how, if at all, decapod crustaceans can be securely anesthetized previous to treating them in physiological experiments or in kitchens.

As a prerequisite for both applications, anesthetization has to be performed without pharmacological tools, which would interfere with the experimental results or the consumers. For this reason four methods were analysed in detail: (a) addition of MgCl_2_ to the holding water (b) cooling (c) bubbling holding water with CO_2_ (d) electric stunning. In addition, the neuronal response to rising temperatures during slow heating was recorded. We implanted electrodes into the CNS of experimental lobsters and crayfish to allow free movements of animals during “normal”behaviour and treatment.

We define complete anesthesia as the absence of differences between unstimulated and externally stimulated signals (signal ratio of one) propagating along nerve fibers and reaching the CNS, including all sensory neuronal responses.

## Material and Methods

### Animals

Crayfish *Astacus* (*Astacus astacus*, and A*stacus leptodactilus*) and American lobsters, *Homarus americanus*, weighing 400–800 gr, were bought from local commercial suppliers (Edelfisch-Kontor Bremerhaven, Deutsche See Bremerhaven), Germany. Young european lobsters *Homarus gammarus* (weighing 15–50 gr) were supplied by the Alfred Wegener Institute, Biologische Anstalt Helgoland, Germany. The animals were kept in aquaria at 7°C previous to the experiments (starting with 7°C) in a day/night cycle of 16/8 hours. The water was exchanged regularly and the animals where fed regularly with fish food. Experiments were announced (and approved) to the Senatorin für Bildung, Wissenschaft und Gesundheit of the state Bremen.

### Preparation

**Fig 1:**
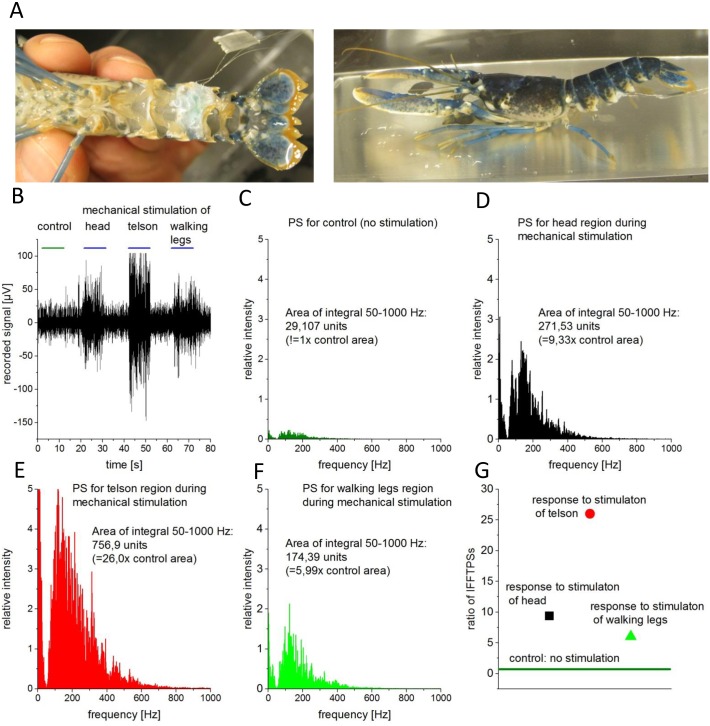
A: Juvenile lobster with implanted hook electrode between the third and fourth abdominal segment. The hook electrode is fixed around the connective between the ganglia. B: Recording trace during control conditions and mechanical stimulation of the head, the telson and the walking legs (blue marker). C-F: Power spectra of the FFTs performed during 5 s of the traces in B during the indicated conditions control (C), head- (D), telson- (E), and leg-stimulation (F). G: The integral of FFTs (black: head; red: telson; green: legs) were divided by the integral of control (without stimulation) to reveal the ratio of integral FFT Power Spectra (IFFTPS). If the ratio is one (green line) the animals were regarded as anaesthetized.

Prior to experimental preparation the animals were partly immobilized by cooling them in ice flakes (0°C) for 30 to 60 minutes. A small U-shaped opening above the ventral side of the connectives between the third and fourth abdominal ganglia was cut with a driller and the epidermis flipped away. Covering muscles were dissected (Fig 1A). The preparation was inspired by the method from Gruhn and Rathmayer [19]. The stability of the anchoring for the hook electrodes was considered as important to the signal quality. A small hook of flat tapped silver wire was used to raise the connectives out of the hemolymph. Remaining hemolymph was sucked off with lab paper. Three (twisted) strands of 75 μm diameter of teflon-coated steel wire (WPI), with length of 75 cm each, were used for two hook electrodes as well as a grounding wire. The teflon at the end of the wires was removed by means of a burning flame; the steel was grinded and cleaned afterwards. The hook electrodes encircled the fused connectives at a distance of a few mm to each other. The ganglionic sheath was not removed. The hooks were insulated from the hemolymph by pouring fast curing silicon (Kwik-cast, WPI) around the hooks and the connectives. As the silicon does not cure fast at low temperatures, a small tube was placed above the silicon, and warm air (RT) blown through it. The third steel wire was placed openly next to the connectives.

The wires were lead out laterally, and the epidermis sheet was flipped back into place, first sealed with a tissue adhesive (Vetbond, 3M) and then superficially covered with glue (Loctite 454, Henkel). To avoid movements of the cables within the animal, the segmental lateral joints were fixed (Loctite).

After the preparation the animals were taken out of the ice, manually stimulated with a glass rod at the antenna and the telson, and their overall activity visually observed. Then they were put back into aquaria with water at the appropriate temperature and were allowed to recover overnight. Fully recovered animals were able to walk around and behave normally (e.g. tail flipping).

### Electrophysiological setup

During the preparation the three wires were connected to an A.C. amplifier (P55, Grass) and the signals bandpath filtered from 3 Hz to 3 kHz, as well as with a 50 Hz line filter. Amplification was ten thousand, digitized (1401 plus, CED) at 16,7 kHz and recorded to hard disk (Spike 2, CED). The signal was used to monitor the signalling of the hook electrodes. After establishment of a sufficient signal, the hooks were embedded in silicon.

Electrical stimulation was performed at the antennae with a stimulator (SD9, Grass) applying a voltage high enough to trigger a visually observable behavioural response of the animal (e.g. movement of appendages, pulling in of eyes, deflection of telson).

### Experiments

During the experiments the animals were placed in an aquarium and kept in place with a clamp (from dorsal, around the carapace). The signal was recorded previous to treatments to reveal a baseline as control. Then the animal was stimulated by (1) touching it with a glass rod at (a) the antenna and eyes, (b) the telson, and (c) the walking legs, (2) by electrical stimulation.

### Treatments

The animals were exposed to one of the following conditions: (1) 10% w/v MgCl_2_*6H_2_O (Merck) was added to the bath. (2) Bubbling of CO_2_ to the aquarium water. The water in the experimental aquarium was aerated with CO_2_ for 15 minutes, until the pH was around pH 5. Then the animal was exposed to the water until animals were anesthetized. The CO_2_ aeriation of the aquarium water was maintained during the course of an experiment. (3) Cooling to 0°C in frozen tap water slurry or to -1.8°C in frozen seawater slurry for 1h. (4) Electric stunning, and (5) slow heating during mechanical and electrical stimulation and previous to exposure to (6) hot water.

#### Electric stunning (4)

We used two different devices: One is constructed for the investigation of electrical stunning of fish (trouts) [20] by the LAVES (Landesamt für Verbrauchersschutz und Lebensmittelsicherheit Niedersachsen, Regional Office for Consumer Protection and Food Security of Lower Saxony). The animal was either transferred into a custom build special aquarium between two (dorsal/ventral) electrodes, fully covered by water and exposed to a DC current of approximately 5 ampere, 20 volts, for 0.5, 1.0, 5.0 or 10 seconds, respectively. The water in the container was either fresh water (*Astacus*), sea water (*Homarus*), or a mixture of both (3g/L; Astacus and Homarus). The second stunning apparatus was a commercially available device (CrustastunTM company, Cambridge, UK) and we followed the manufacturers operating procedures.

During treatment and recovery, animals were stimulated regularly. Animals exposed to hot water were stimulated electrically on the antennae every 15 seconds until no electrophysiological signal was detected anymore. Animals exposed to conditions 2, 4, and 5, were stimulated regularly at least one hour following the treatment. An animal was categorized “dead” if no signals could be recorded from the nervous system and the animal looked dead by means of visual inspection several hours after the treatment.

### Electrophysiological signal processing

Additionally to the visual inspection of the recorded signals (e.g. action potentials present/not present) the signal was processed using the Fast Fourier Transformation (FFT) within the recording software and the power spectrum was calculated for periods of five seconds during rest and during stimulation. The integral between 50 and 1000 Hz of the power spectrum was calculated as a measure of neuronal activity (integral FFT power spectra IFFTPS) (Fig 1B–1G). An animal was categorized as “anaesthetized” when the ratio of the IFFTPS (during/before stimulation) was approaching one. For untreated animals the ratio of IFFTPS during/before stimulation was in the range between three and thirty.

## Results

The recorded electrophysiological signal traces from implanted electrodes in the CNS of freely moving lobster and crayfish showed significant differences between resting and/or active/stimulated states and a reliable signal to noise ratio (Fig 1). Analysis of the signals by means of action potential counting was not used as many recordings displayed a high number of summated action potentials. Instead, the integrals of the fast fourier transformation power spectra (IFFTPS) were used to calculate the ratio between the stimulated and the basal activity (see Methods section). The full area of the bins in the power spectrum histogram (Fig 1B–1G) was calculated and the ratio between baseline and stimulation was in the range between 3 and 30. The animals were considered to be anesthetized when the ratio of IFFTPS was close to 1 (Fig 1G).

### MgCl_2_

**Fig 2:**
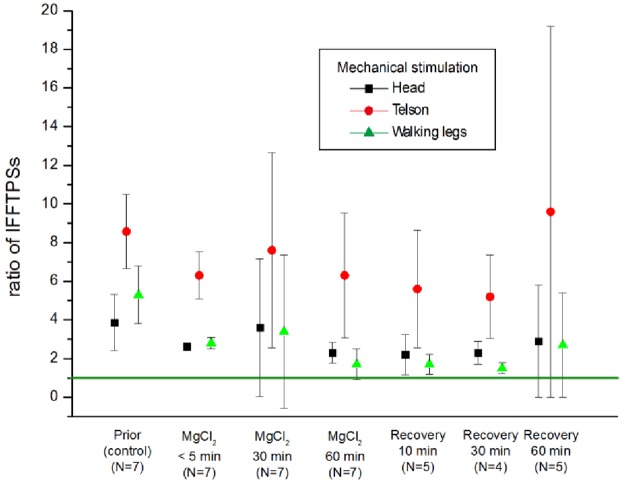
Relative response, the integral of FFT power spectra IFFTPS ratios (stimulated/not stimulated), during control conditions and treatment with 10% MgCl_2_ after 5 min, 30 min and 60 min and corresponding recovery (washout). Responses to mechanical stimulation of head (black), telson (red) and legs (green) are shown.

MgCl_2_ is long known to anaesthetize/immobilize marine invertebrates [21,22] and was applied in crustaceans [23], but in our experiments exposure to 10% MgCl_2_ failed to produce an anaesthetic effect in lobster even after one hour. The calculated electrophysiological response showed no effect of MgCl_2_ application (Fig 2).

### Cooling 0°C and cooling -1,8°C

**Fig 3:**
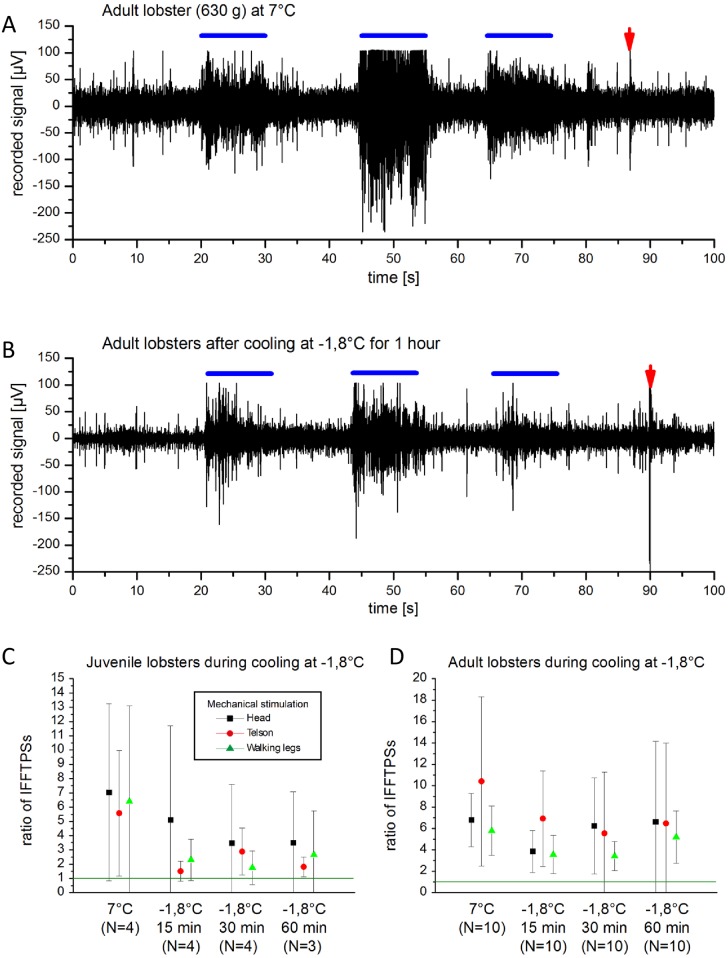
Recording traces of adult lobster (A) at 7°C (control) and (B) after 1h in frozen seawater slurry (-1,8°C). Marking lines reflect mechanical stimulation at the head, telson and legs, respectively. Red arrow marks electrical stimulation. IFFTPS ratios of (C) juvenile and (D) adult lobster during 15 min, 30 min and 60 min in frozen seawater.

Cooling of animals in tap water ice (0°C) caused only minor reduction of neuronal responses. Fig 3 summarizes the results from our experiments of cooling lobsters in frozen sea water slurry (-1.8°C) where signal transduction was still detectable after 1h of cooling. Signal to basal activity ratio (noise) remained nearly unchanged during experimentation. Anaesthesia in terms of the absence of response to mechanical and electrical stimulation was not seen after 60 min of cooling juvenile (N = 5) and adult lobsters (N = 5) to -1.8°C in seawater. Experiments with crayfish cooled to 0°C in fresh water slurries yielded similar results (no anaesthesia after one hour, data not shown). Visual inspection of the crayfish revealed a minor degree of responsiveness/stress perception, e.g. retraction of eye stalks upon stimulation.

### CO_2_

**Fig 4:**
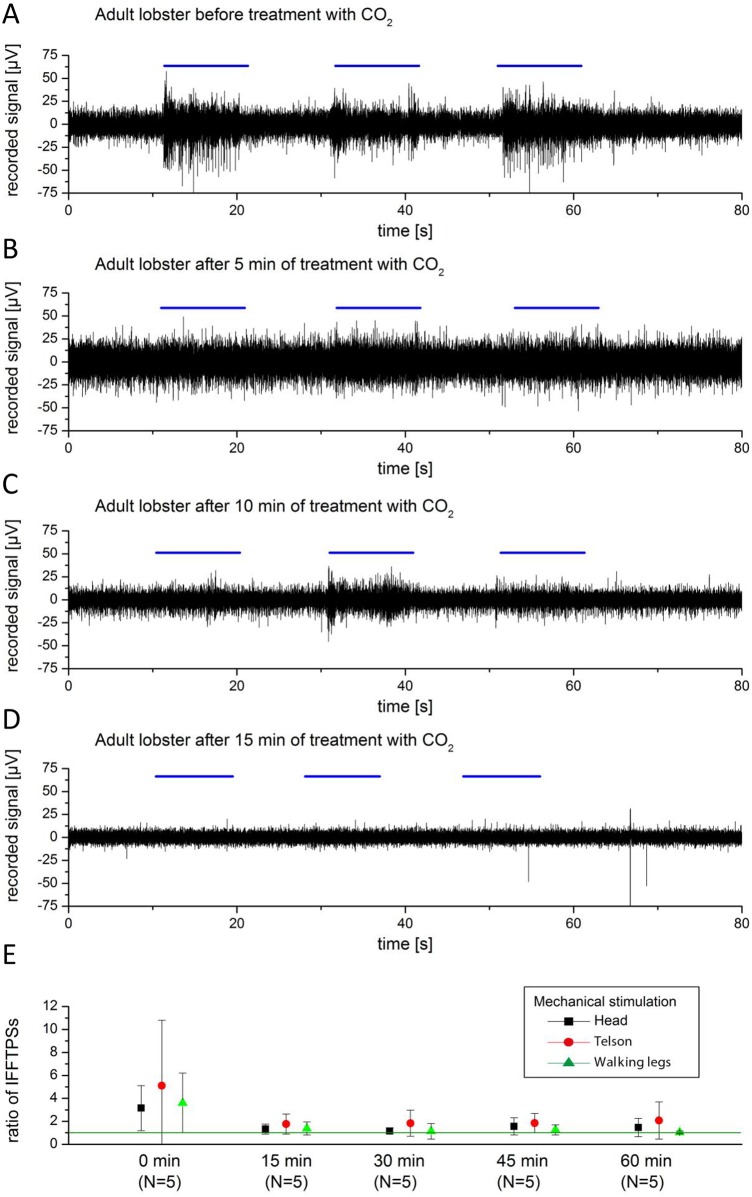
A: Recording traces at time 0 min, 5 min, 10 min and 15 min (A, B, C, D) in seawater bubbled with CO_2_ (pH ~5). E: IFFTPS ratios after 0, 15, 30, 45, and 60 min of CO_2_ exposure.

Bubbling of the aquarium water with CO_2_ gas lowers the water pH from 8 to approximately pH 5. This strong change of seawater pH causes aversive and agitated behaviour in animals trying to avoid the CO_2_ gas before signs of anaesthesia were observed after approximately 10 to 45 minutes in juvenile (N = 5) and adult lobster (N = 5). Exposure of lobsters to CO_2_ gas induces anaesthesia (Fig 4). Recording traces indicate absence of stimulus-related responses whereas the integral of the power spectra attained stimulus/control (IFFTPS) ratios close to 1, corresponding to our definition of anaesthesia (Fig 4). The aspect of torpid animals can best be described as “slack” with hanging appendages. After 45 min anaesthesia was reached in all experimental animals, which was fully reversible after recovery overnight.

### Electric stunning

**Fig 5:**
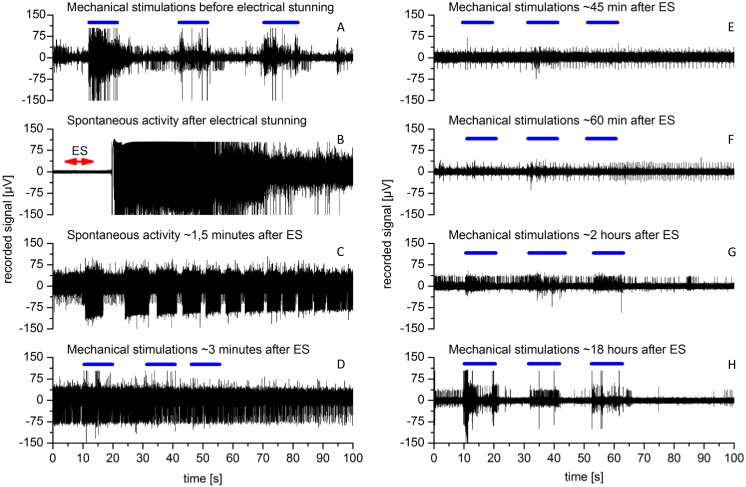
Recording traces previous to (A) shortly after (B) and after electrical stunning (1 min, 3 min) (C, D) and with mechanical stimulation and recovery after up to 18 h (E-H).

**Fig 6:**
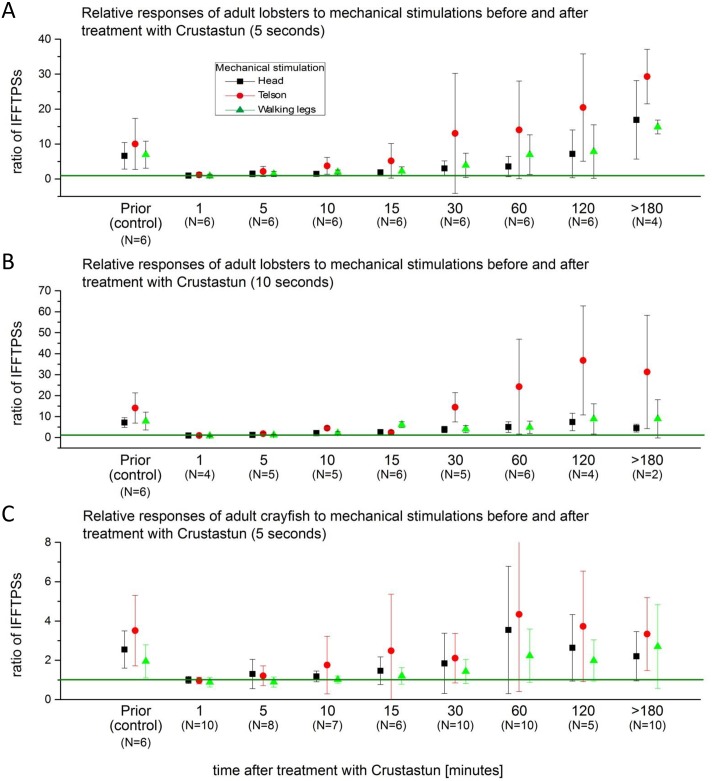
IFFTPS ratios during different time points before and after electrical stunning. A: Adult lobster stunned with Crustastun during 5 s. B: Adult lobster stunned with Crustastun during 10 s. C: Adult crayfish stunned with Crustastun (5 s).

Electric stunning was performed using two different devices (see methods). Treatment with both instruments yielded similar results in terms of the recorded electrophysiological traces of the activity in the CNS of electro-stunned lobster and crayfish. Shortly after electrical stunning, the visual appearance of the subjects was that of anaesthetized animals. Muscle tonus appeared tense, with frequent retraction of eye stalks. This stiffness faded within minutes after which the animals appeared slack and numb, and further stimulations failed to trigger reflexes. Recovery was slow and required several hours. The electrophysiological recordings showed a strong permanent activity in the CNS during the phase of muscle tension, which declined as muscles slacked. During the high activity phase, no discrimination of external stimuli processing could be achieved as the primary electrophysiological activity after stunning covered the whole potential spectrum. Regarding our definition of anaesthesia, the animals were fully anaesthetized, despite seizure like electrical signalling within the CNS. These pronounced signals diminished throughout the duration of the experiment until 10–60 min into the recovery phase, when signal discrimination started to increase again (Fig 5). All animals electro-shocked with the “LAVES” device showed a seizure like increase of neuronal activity, independent of the applied stunning duration (up to 10 s). The “Crustastun” device possess two adjustments for 5 s and 10 s stunning duration. The difference between the two stunning steps was a delay in recovery after 10 s of stunning compared to the 5 s lasting treatment (compare Fig 6A and 6B). The Crustastun device induced epilepsy-like seizures in lobsters, whereas crayfish showed only occasional seizures after stunning (Crustastun 5 seconds) (regularly with the LAVES instrument).

### Slow heating

**Fig 7:**
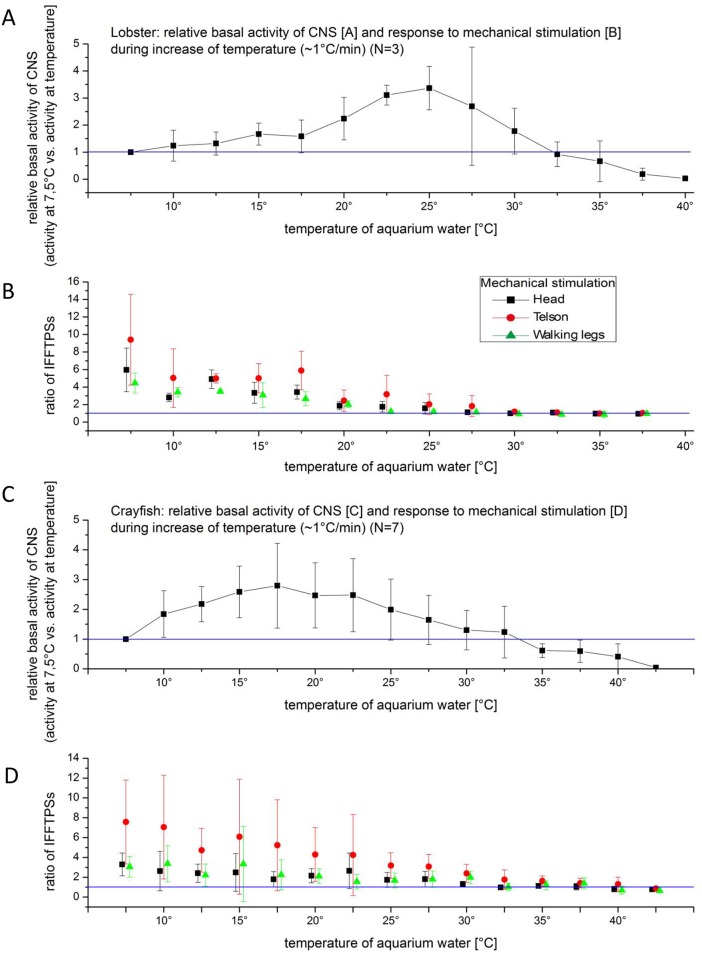
A: Adult lobster CNS activity without stimulation during slowly heating (1°C/min) of water. IFFTPS ratios of recording traces baselines. B: IFFTPS ratios of adult lobster during slow heating (1°C / min) starting from 7°C up to 45°C during stimulation at head, telson and legs. C: Adult crayfish CNS activity without stimulation during slowly heating (1°C/min) of water. IFFTPS ratios of recording traces baselines. D: IFFTPS ratios during slow heating (1°C / min) starting from 7°C up to 45°C during stimulation at head, telson and legs.

“Urban legend” of slow heating applied to prepare frogs prompted us to embark on similar experiments with electrode implanted lobsters (N = 3) and crayfish (N = 7). The recorded baseline signal was compared to responses triggered by external stimuli, following the procedures of the other experiments of this series. Slowly incrementing water temperature from 7°C to 40°C (~1°/ min) elicited a similar response in both, lobster and crayfish. The signal to baseline ratio was highest between 7°C and 20°C and with increasing temperature above 32°C the ratio decreased to one. The animals were killed when temperatures were slowly raised without any apparent signs of stress or movements. The maximum basal activity in crayfish was recorded at 25°C and at 17.5°C in lobster, which may reflect their temperature optima (Fig 7).

### Exposure to hot water

**Fig 8:**
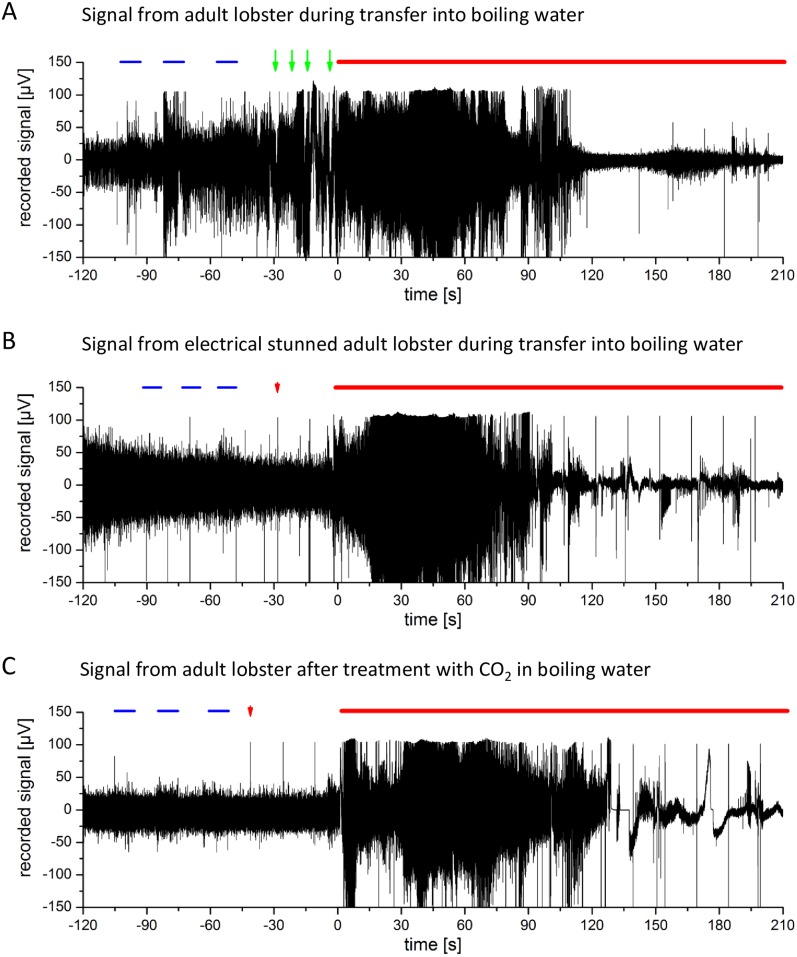
A: Lobster CNS recordings before and during exposure to boiling hot water. Blue lines mark mechanical stimulations. Red line marks the hot water treatment. Green arrows indicate tail flips. B: Following electrical stunning: Lobster CNS recording trace before and during application of hot water. Red line marks treatment. Red arrows indicate electrical stimulation before and during treatment (only first one indicated; regular stimulation artefacts in trace app. all 15 seconds). C: Following CO_2_ treatment: Lobster CNS recording trace before and during application of hot water. Red line marks treatment, red arrow begin of electrical stimulation.

In all control animals an increase in motility and therefore electrophysiological signals was observed during the handling procedures previous to exposure to hot water, including some incidents of vigorous tail flips indicating escape response. When the animals were exposed to the boiling water (Fig 8) movements and aversive behaviour stopped. In disagreement with previous results [1], neither enhanced motile activity nor tail flipping were observed in animals placed in hot water. However, we recorded a strong increase of electrophysiological signals and an increase in the integrals of FFTs, which abated after roughly 30 s to 150 s pending on animal size (Fig 5). The decline of electrophysiological signals following transfer of the animals from 7°C water to boiling water was at 46.9 s ± 11.6 s for juvenile lobster (23,1 g ± 8,2 g; N = 5) and at 154.7 s ± 30.3 s for adult lobster (635 g ± 78 g, N = 5).

### Exposure to hot water following previous treatments

**Fig 9:**
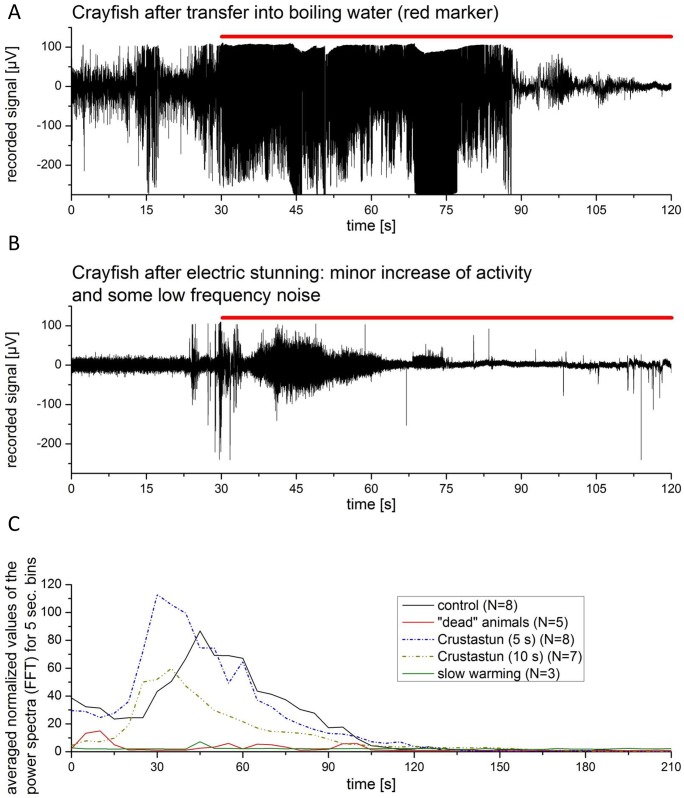
A: Crayfish CNS recording traces before and during application of hot water. Red line marks treatment (100°C). B: Following electrical stunning: Crayfish CNS recording traces before and during application of hot water. Red line marks treatment. C: Averaged FFTPS of adult lobsters in hot water without prior treatment (control), and after treatment electrical stunning with Crustastun, slow warming, and (visually) dead animals (did not recover after surgery).

For the group of the adult lobsters, we did not observe differences regarding the end of electrophysiological signals between pre-treated animals that had undergone anaesthesia treatment. The integral of FFT power spectra IFFTPS revealed no reduction of signals after “anaesthesia” (Fig 9).

**Fig 10:**
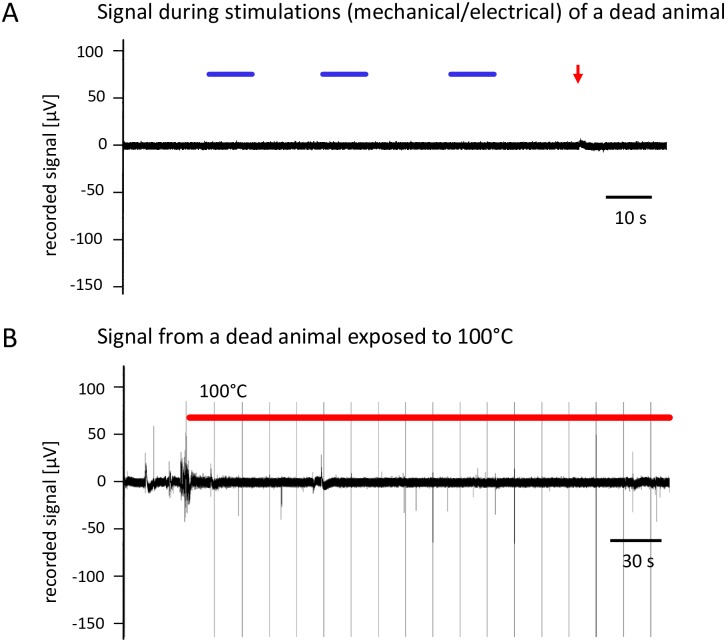
A: Control recording trace during stimulation of a dead lobster. B: Control recording of a dead animal in hot water. Touching of animal during transfer to hot water cause some distortions.

We further recorded “signals” of recently dead animals (N = 3) (Fig 10) as control during mechanical stimulation and exposure to hot water. We found exposure to hot water to induce some electrical signals generated from undefined electrical processes during cooking.

Crayfish stunned with Crustastun (5 s) (N = 5) showed a minor increase (compared to control) of the recorded electrophysiological signals after transfer into boiling water (Fig 9).

In stunned lobster we found a reduction or late onset of the increase of electrophysiological signals during the first 20 s after exposure to hot water, which was the only detectable difference between treated and untreated animals (Fig 5B).

Autotomy was not observed (discarding of limbs due to neuronal processing). These results contradict previous outcomes reported in the literature [1,3].

Supplementary data can be found in S1 Tables.

## Discussion

Avoiding injury, also conceived as “nociceptive reflex”, is the most natural behavior seen in healthy individuals across all animal groups. Regardless of whether and how “pain” can be defined (or not) in invertebrates [24,25], we consider nociceptive reflexes and pain as two sides of the same coin. We defined anesthesia as the neuronal response to external stimuli to be not distinguishable to unstimulated controls and therefore the ratio of integrals of power spectra (IFFTPS) of both states as cipher one. The neuronal excitation of an individual can hence be determined as an increased basal activity of neuronal signals in the brain, occurring without external stimulation and tractable as “noisy signaling” in the CNS.

Slowly rising water temperatures (1°C/min) did not cause unusual excitation of CNS electrical activities until reaching about 30°C, where the CNS did not show any electrical activity to external stimuli (mechanical and electrical). Prior to this point the animals did not show obvious signs of stress or escape behavior (we did not record or visually observed any tail flips).

Applying this concept to the methods to sacrifice a lobster or crayfish in the present experimental series, we were surprised to find that slow warming is a usable method. Crustacea possess temperature sensitive neurons [26–28] and are able to discriminate temperature changes and to react adequately [29]. Behaviours are associated to avoid extremes as well.

Larval crabs can avoid colder water but upon warming the larvae are induced to swim to greater depths [30,31,32]. Lobster postlarval stages show active behaviour prefering optimum settlement temperature around 16°C, in their natural environment [33].

Fast temperature shifts are presumably a less dramatic part of the natural environment of lobsters, *Astacus* or aquatic molluscs compared to terrestrial environments [34]. Thus, no adequate physiological response of adult animals to this stimulus may exist, despite the fact, that crayfish may be exposed naturally to larger temperature changes. In estuarine environments lobsters and crayfish may experience major temperature variations with tide and sun exposure.

The presence of epileptic like seizures covered all incoming sensory inputs induced by stunning, leading to a kind of excited anesthesia. Epilepsy-like seizures occur in chicken [35], sheep [36] and pigs [37] subjected to electrical stunning, and may be a general consequence of this treatment. The neuronal excitation during stunning is so intense that incoming signals could not be discriminated from all other signals. Excitation of many/all neurons leads to paralysis. Using a thermal imager camera (data not shown), we noticed however that animals subjected to stunning with Crustastun were severely heated, producing body surface temperatures with hot spots of >40°C. This sudden heat production during stunning may well have caused a similar result like the heating experiments.

Cooling induces no anesthesia in our experimental groups but might work in species thermally adapted to high ambient temperatures (e.g. *Litopenaeus vannamei* which is kept at 30°C in aquaculture farms). It has to be kept in mind that cooling itself might stress crustaceans [28]. An acute temperature change from 21°C to 5°C seems to inhibit a neuronal response in prawn (*Macrobrachium rosenbergii*) [38] indicating species specific differences in response to cooling.

Neuronal changes induced by cooling may depend on the starting conditions and adaptation of the animals. This would be an interesting topic in a follow up study and would extend the scope of this work.

10% MgCl_2_ has been successfully used to induce anesthesia in many marine animals [21,22,39,40] including some crustaceans [23,41], but 10% MgCl_2_ exposure did not slumber decapods within one hour of bath exposure. How a 20% or 50% MgCl_2_ treatment would be responded is unclear and may be successful in inducing anesthesia.

CO_2_ exposure has good sedative or anesthetic potential in fish [42,43] or rodents [44] with required action times in the range of minutes. CO_2_ has been shown recently to inhibit synaptic transmission in crabs and crayfish very rapidly [45], by in part interacting with glutamate receptors independent of pH [46]. Exposure of lobster and crayfish to CO_2_-acidified water was only partially successful for anesthesia, as pH values lower than 5 units seemed potentially stressful for the animals that appeared restless and agitated previous to anesthesia.

## Conclusion

MgCl_2_ (10%) as well as cooling in tap water (0°C) and sea water (-1,8°C) does not anaesthetize lobster and crayfish. CO_2_ is efficient to anaesthetize lobster and crayfish but due to low pH in water is stress full previous to achieved anaesthetization to the animals. A feasible way to sacrifice lobsters is to slowly raise the water temperature (1°C min^-1^), as all electrical activities in the CNS cease at temperatures above ~30°C, whereas below this temperature the animals do not show signs of stress or escape behavior (e.g. tail flips) in the warming water. Electrical stunning induces epileptiform seizures but paralyses the animals and leads to a reversible decline of nerve system activity after seizure.

Electric stunning or slowly warming just before preparation may meet ethical expectations regarding anesthesia and to sacrifice crustaceans.

## Supporting Information

S1 Tables (PDF)
