# Supplementary material for: Electrophysiological Investigation of Different Methods of Anesthesia in Lobster and Crayfish
Source: PLoS One. 2016 Sep 19;11(9):e0162894. doi: 10.1371/journal.pone.0162894 (PMC5028027; doi:10.1371/journal.pone.0162894)
Supplement: S1 Tables — (PDF) [file pone.0162894.s001.pdf]

Tabellen

Tabelle 1: Auswertung Elektrobehandlung von Hummern und Flusskrebsen mit Gerät „Crustastun“								
	„Epileptische Phase“ nach Behandlung mit Crustastun im Nervensystems			Aktivität nach Überführung in 100°C			Visuell wahrnehmbare Zuckungen des Tieres nach Überführung in 100°C	
	Ja	Nein	Nicht auswertbar	Erst kein Anstieg, dann starker Anstieg	Nur geringer oder kein Anstieg	Nicht auswertbar wegen zu vieler Störsignale	Ja	Nein
Hummer Stufe 1	12	4		7	0	1	7	1
Hummer Stufe 2	9	1		7	1	0	3	5
Flusskrebs Stufe 1	6	18	0	3	12	0		
Flusskrebs Stufe 2	0	5	2	5	1	1		

Tabelle 2: Große Hummer und Flusskrebse nach Betäubung mit Crustastun und Überführung in kochendes Wasser: Fläche unter Kurve einer FFT (Fast Fourier Transformation) bis physiologisch definierter Nullpunkt erreicht ist
Hummer: t-Test: keine signifikanten Unterschiede bei Hummern, daher Daten zusammengefasst
Flusskrebse: t-Test: signifikante Unterschiede (P=0,05) zwischen den Gruppen

	Überführung in 100°C nach	N=	Mittelwert	SD
Hummer Kontrolle (aus Zwischenbericht)	1 min	8	3504,4	1175,0
Hummer Stufe 1	1 min	4	2661,19	1111,26
	5 min	4	3053,74	928,03
Hummer Stufe 2	1 min	3	1281,06	860,13
	5 min	5	3766,55	568,99
Hummer Stufe 1 zusammengefasst	1 und 5 min	8	2857,57	970,76
Hummer Stufe 2 zusammengefasst	1 und 5 min	8	2834,49	1432,16
Flusskrebse Kontrolle	1 min	5 von 5	2355,6	916,7
Flusskrebse Stufe 1	1 min	12 von 15	518,37	334,25
Flusskrebse Stufe 2	1 min	5 von 7	345,9	307,67
Flusskrebse nach langsamer Erwärmung		1 von 7 (Aussetzer des Verstärkers und hochfrequente anhaltende Störsignale)	42,84	

Tabelle 3: Zeitraum bis Erreichung des definierten physiologischen Nullwerts (Fläche unter Kurve einer FFT) nach Überführung in 100°C Wasser. Kein signifikanter Unterschied bei Hummern; signifikanter Unterschied bei Flusskrebsen zwischen Kontrolle und mit Crustastun behandelten Tieren (p=0,5).			
	N=	Mittelwert [Sekunden]	SD
Hummer Kontrolle (aus Zwischenbericht)	8	154,7	30,3
Hummer Stufe 1	8	145,38	46,73
Hummer Stufe 2	8	153	37,13
Flusskrebse Kontrolle	5	79	16,7
Flusskrebse Stufe 1	12 von 15	45,91	12,1
Flusskrebse Stufe 2	5 von 7	41,2	8,04

Tabelle 4 : Langzeitüberleben bzw. Letalität nach Betäubung mit Crustastun bei Hummer *Homarus americanus*, Flusskrebs *Astacus leptodactylus*, Strandkrabbe *Carcinus maenas*, Taschenkrebs *Cancer pagurus*			
	N=	Erholung des Tieres	Tier tot
Hummer Stufe 1	7	5	2
Hummer Stufe 2	3	1	2
Flusskrebse Stufe 1 (mit Ableitung)	8	6	2
Flusskrebse Stufe 1 (ohne Ableitung)	10	9	1
Flusskrebse Stufe 2 (ohne Ableitung)	10	9	1
Strandkrabben Stufe 1	10	9	1
Strandkrabben Stufe 2	10	5	5
Taschenkrebse Stufe 2	6	3	3

Tabelle 5: Relative Reizweiterleitung bei Großen Hummern *Homarus americanus* nach Behandlung mit Crustastun							
	Reizort	Stufe 1 N=	Mittelwert Stufe 1	SD	Stufe 2 N=	Mittelwert Stufe 2	SD
Kontrolle/ vor Behandlung	Kopf	7	6,61687	3,80022	6	7,15238	2,33984
	Telson	7	10,01996	7,31388	6	14,07911	7,23287
	Schreitbeine	7	6,9651	3,87209	6	7,82724	4,23406
1 min	Kopf	6	0,96834	0,16334	4	0,93354	0,1123
	Telson	6	1,18488	0,72459	4	0,98151	0,24501
	Schreitbeine	6	0,84533	0,48763	4	0,77935	0,18813
5 min	Kopf	5	1,45462	0,8825	5	1,24339	0,37149
	Telson	5	2,14939	1,47097	5	1,84199	0,50129
	Schreitbeine	5	1,42659	0,75499	5	1,20914	0,2859
10 min	Kopf	6	1,46343	0,30773	5	2,15456	0,80282
	Telson	6	3,74171	2,39392	5	4,50325	0,92887
	Schreitbeine	6	1,97161	0,57543	5	2,09342	0,67808
15 min	Kopf	6	1,88888	0,65542	6	2,5734	1,35401
	Telson	6	5,17317	4,93665	6	2,42768	0,906
	Schreitbeine	6	2,25134	1,20326	6	6,13331	1,41403
30 min	Kopf	6	3,0244	2,15855	5	3,82702	1,52699
	Telson	6	13,0525	17,19273	5	14,45613	7,019
	Schreitbeine	6	3,91273	3,47846	5	4,0314	1,68911
60 min	Kopf	6	3,56777	2,92068	6	4,99978	2,57315
	Telson	6	14,04505	13,95111	6	24,26768	22,62707
	Schreitbeine	6	6,95889	5,67835	6	4,91068	2,91615
120 min	Kopf	6	7,17495	6,8431	4	7,41114	4,13192
	Telson	6	20,43875	15,35562	4	36,78235	25,99935
	Schreitbeine	6	7,83015	7,65571	4	8,85981	7,21296
>180 min	Kopf	4	16,90759	11,24219	2	4,48604	1,89529
	Telson	4	29,30172	7,8099	2	31,27318	26,97802
	Schreitbeine	4	14,89597	1,97428	2	8,94198	9,11655

Tabelle 6: Relative Reizweiterleitung bei Flusskrebsen nach Behandlung mit Crustastun (Stufe 1)				
	Reizort	N=	Mittelwert	SD
Kontrolle/Vor Betäubung	Kopf	10	2,54701	0,94605
	Telson	10	3,51039	1,79286
	Schreitbeine	10	1,94862	0,84157
1 min	Kopf	10	0,99991	0,17931
	Telson	10	0,96979	0,16831
	Schreitbeine	10	0,88345	0,24294
5 min	Kopf	8	1,30195	0,74384
	Telson	8	1,21345	0,50588
	Schreitbeine	8	0,89403	0,25631
10 min	Kopf	7	1,18077	0,27611
	Telson	7	1,75787	1,46733
	Schreitbeine	7	1,01557	0,1808
15 min	Kopf	6	1,46745	0,70383
	Telson	6	2,48228	2,8771
	Schreitbeine	6	1,20199	0,42054
30 min	Kopf	10	1,84334	1,53668
	Telson	10	2,10669	1,25877
	Schreitbeine	10	1,43311	0,61627
60 min	Kopf	10	3,54455	3,24134
	Telson	10	4,33641	3,93382
	Schreitbeine	10	2,22954	1,35643
120 min	Kopf	5	2,63422	1,69089
	Telson	5	3,72533	2,81147
	Schreitbeine	5	1,98696	1,0481
>180 min	Kopf	10	2,20397	1,25507
	Telson	10	3,33702	1,85711
	Schreitbeine	10	2,69788	2,12931

Tabelle 7 – Daten des Experiments mit langsamer Erwärmung des Hälterungswassers bei Hummern *Homarus americanus*						
Temperatur	Stimulationsort	N=	Mittelwert relative Aktivität bei mechanischer Stimulation	Standard-abweichung	Mittelwert relative Grundaktivität	Standard-abweichung
7,5°C	Kopf	3	5,95675	2,48738	1	
	Telson	3	9,41016	5,19393		
	Schreitbeine	3	4,45117	1,15307		
10°C	Kopf	3	2,81206	0,47839	1,2385	0,56781
	Telson	3	5,03516	3,34104		
	Schreitbeine	2	3,41786	0,52094		
12,5°C	Kopf	2	4,90564	1,057	1,31995	0,42433
	Telson	2	4,98303	0,53979		
	Schreitbeine	2	3,49521	0,21766		
15°C	Kopf	2	3,33678	1,19561	1,667	0,40447
	Telson	2	5,00278	1,66674		
	Schreitbeine	2	3,07702	1,40982		
17,5°C	Kopf	2	3,41708	0,79024	1,582	0,6067
	Telson	2	5,8766	2,20584		
	Schreitbeine	2	2,66137	0,81508		
20°C	Kopf	3	1,86676	0,47273	2,238	0,7863
	Telson	3	2,43857	1,23813		
	Schreitbeine	2	2,01079	0,39679		
22,5°C	Kopf	2	1,74268	0,60084	3,1075	0,36699
	Telson	2	3,16929	2,16215		
	Schreitbeine	2	1,19027	0,14613		
25°C	Kopf	3	1,57588	0,66012	3,367	0,80327
	Telson	3	2,01688	1,19468		
	Schreitbeine	3	1,17319	0,173		
27,5°C	Kopf	2	1,10786	0,24546	2,6915	2,18425
	Telson	2	1,82017	1,2053		
	Schreitbeine	2	1,12916	0,12196		
30°C	Kopf	3	0,9966	0,06491	1,77655	0,84209
	Telson	3	1,18046	0,27598		
	Schreitbeine	3	0,91887	0,07556		

32,5°C	Kopf	3	1,07375	0,16804	0,921	0,45396
	Telson	3	1,09141	0,15336		
	Schreitbeine	3	0,87904	0,14513		
35°C	Kopf	3	0,95873	0,0367	0,66145	0,75314
	Telson	3	0,96648	0,16531		
	Schreitbeine	3	0,85914	0,38517		
37,5°C	Kopf	3	0,93813	0,12291	0,186	0,22203
	Telson	3	1,018	0,08529		
	Schreitbeine	3	0,95627	0,23274		
40,0°C	n/a	1	n/a	n/a	0,0258	

Tabelle 8 – Daten des Experiments mit langsamer Erwärmung des Hälterungswassers bei Flusskrebsen *Astacus leptodactylus*						
Temperatur	Stimulationsort	N=	Mittelwert relative Aktivität bei mechanischer Stimulation	Standard-abweichung	Mittelwert relative Grundaktivität	Standard-abweichung
7,5°C	Kopf	7	3,2976	1,15083	1	
	Telson	7	7,57741	4,21973		
	Schreitbeine	7	3,0518	1,05094		
10°C	Kopf	6	2,61873	1,989	1,84107	0,78591
	Telson	6	7,05488	5,21857		
	Schreitbeine	6	3,36806	1,81376		
12,5°C	Kopf	7	2,40542	0,91866	2,17651	0,58957
	Telson	7	4,72813	2,2072		
	Schreitbeine	7	2,22291	1,13568		
15°C	Kopf	7	2,48837	1,89566	2,58886	0,86635
	Telson	7	6,08304	5,79006		
	Schreitbeine	7	3,33734	3,7962		
17,5°C	Kopf	7	1,79956	0,78349	2,79814	1,42503
	Telson	7	5,22648	4,58781		
	Schreitbeine	7	2,25043	1,5184		
20°C	Kopf	7	2,15972	0,71226	2,47006	1,09267
	Telson	7	4,29158	2,70721		
	Schreitbeine	7	2,11195	0,72949		
22,5°C	Kopf	7	2,65134	1,79114	2,476	1,22877
	Telson	7	4,24591	4,08817		
	Schreitbeine	7	1,55966	0,72585		
25°C	Kopf	7	1,7383	0,746	1,99027	1,023
	Telson	7	3,17681	1,29352		
	Schreitbeine	7	1,6762	0,74494		
27,5°C	Kopf	7	1,80843	0,79273	1,64543	0,82947
	Telson	7	3,07544	1,21615		
	Schreitbeine	7	1,80622	0,80781		
30°C	Kopf	7	1,31867	0,14001	1,30317	0,66265
	Telson	7	2,39685	0,91911		
	Schreitbeine	7	1,97958	0,6383		
32,5°C	Kopf	7	0,97326	0,24314	1,23463	0,86714
	Telson	7	1,76355	0,97823		
	Schreitbeine	7	1,02392	0,36579		
35°C	Kopf	7	1,12433	0,2506	0,61526	0,22906
	Telson	7	1,62338	0,52153		
	Schreitbeine	7	1,20865	0,45811		
37,5°C	Kopf	6	1,04783	0,32756	0,59362	0,37312

	Telson	6	1,40833	0,48089		
	Schreitbeine	6	1,3833	0,56676		
40°C	Kopf	6	0,81441	0,28528	0,40968	0,43481
	Telson	6	1,29649	0,71338		
	Schreitbeine	6	0,69249	0,40812		
42,5°C	Kopf	2	0,7898	0,23097	0,043 (N=1)	
	Telson	2	0,864	0,19896		
	Schreitbeine	2	0,64159	0,0605		
